# Response to letter regarding ‘impact of time from blood specimens collection to centrifugation on the diagnosis of gestational diabetes mellitus’

**DOI:** 10.1080/07853890.2026.2670062

**Published:** 2026-05-07

**Authors:** Binbin Yin, Bo Zhu

**Affiliations:** Department of Laboratory Medicine, Women’s Hospital, Zhejiang University School of Medicine, Hangzhou, Zhejiang, People’s Republic of China

**Keywords:** Gestational diabetes mellitus, laboratory quality, accurate diagnosis

We would like to express our sincere gratitude to Dr. Tang for his comments on our study [1]. As laboratory staff, we are delighted to discuss the details of the study with clinicians, as this highlights the differences and complementarities between laboratory and clinical perspectives. Dr. Tang emphasized the clinical objective of accurate diagnosis. Our study, grounded in the laboratory quality management system, aims to optimize workflows and ensure test quality [2].

## 1. Regarding the type of blood collection tube

We acknowledge that the type of blood collection tube (particularly whether it contains a glycolysis inhibitor) affects the stability of blood glucose [3]. However, this study focuses on real-world scenarios: currently, most hospitals routinely use gel-barrier serum tubes without glycolysis inhibitors to balance costs or to facilitate the sharing of samples for multiple tests [4]. The introduction of specialized tubes would disrupt workflows and increase the volume of blood required, limiting feasibility. Therefore, in a real-world setting, all samples in this study were collected using BD Vacutainer gel-barrier serum tubes (without added glycolysis inhibitors). This has been clearly stated in the ‘Methods’ section to accurately reflect the current state of specimen handling in clinical practice.

## 2. Regarding the ‘reclassification’ data analysis

Dr. Tang suggested analyzing the specific proportion of individuals who transitioned from positive to negative. However, in a real-world retrospective analysis, the available data do not permit the precise calculation of the number of gestational diabetes mellitus (GDM) cases that transitioned from positive to negative due to centrifugation delays. The primary objective of this study is to assess the system-wide impact of prolonged pre-analytical processing time on population-level incidence, rather than to evaluate individual cases. We fully acknowledge that ‘diagnostic reclassification’ at the individual level holds significant clinical importance. However, this issue requires investigation through prospective intervention studies [5].

## 3. Regarding the classification scoring system and continuous time variables

The choice of a categorical scoring system in this study was based on two considerations: (1) consistency with routine laboratory quality monitoring indicators (such as specimen processing rates within 30, 60, and 90 min), facilitating sample management; and (2) providing laboratories with an intuitive ‘risk warning signal’, where a high score indicates a risk of delay for the entire batch of samples. This design assists laboratories in establishing feasible ‘separation time’ quality control targets for the entire process. Furthermore, Table 2 in the study presents trends in average glucose levels [2] across different processing times, clearly demonstrating the impact of delays on results – a factor critical for diagnostic accuracy, particularly in women with glucose levels near the cut-off value.

In summary, this discussion has enriched our understanding of pre-analytical factors in the diagnosis of GDM. This study is grounded in real-world practice: delayed sample centrifugation can affect the diagnosis of GDM. We share the same objective as Dr. Tang: accurate diagnosis of GDM. We hope this study will serve as a bridge, encouraging clinicians to pay attention to pre-analytical sample processing and motivating laboratories to set stricter standards, thereby better supporting clinical decision-making.

## Data Availability

Data sharing does not apply to this article because no new data was generated or analyzed in this study.
